# Collider stratification bias II: magnitude of bias

**DOI:** 10.1093/aje/kwae255

**Published:** 2024-08-06

**Authors:** Hailey R Banack, Elizabeth Rose Mayeda, Matthew P Fox, Ashley I Naimi, Brian W Whitcomb

**Affiliations:** Epidemiology Division, Dalla Lana School of Public Health, University of Toronto, Toronto, ON M5T 3M7, Canada; Department of Epidemiology, UCLA Fielding School of Public Health, Los Angeles, CA 90095, United States; Department of Epidemiology and Department of Global Health, Boston University, Boston, MA 02118, United States; Department of Epidemiology, Emory University, Atlanta, GA 30322, United States; Department of Biostatistics and Epidemiology, University of Massachusetts Amherst, Amherst, MA 01003, United States

**Keywords:** bias, collider stratification bias, selection bias, directed acyclic graphs, *AJE* classroom

A previous *AJE* Classroom discussed the structure of collider stratification bias and presented applied examples of how it can be introduced by conditioning via restriction or stratification on a collider.^1^ Collider stratification bias results in spurious associations that lack both internal and external validity; biased estimates are not reflective of any causal relationship for any population, even the population that produced the estimates being considered.^2^ To further examine the concepts presented in the first article, in this companion article, we shift our focus from the structure of collider stratification bias to the magnitude of bias introduced by conditioning on a collider. In this article, our goal is to examine the factors that drive the magnitude of collider stratification bias and quantitatively assess the degree to which collider stratification bias may cause effect estimates to deviate from the truth.

Various epidemiologic paradoxes have been proposed to have collider stratification bias at their root, such as the birthweight paradox and the obesity paradox.^3^^,^^4^ In these scenarios, conditioning on a collider meaningfully alters study results, such that an apparently harmful exposure (eg, smoking, obesity) appears protective against death as a result of conditioning on a collider (eg, low birthweight, chronic disease). Given that both obesity and smoking are widely accepted as risk factors for disease, results demonstrating a beneficial effect are considered unexpected or “paradoxical.”

## Quantifying the magnitude of bias

**Figure 1:**
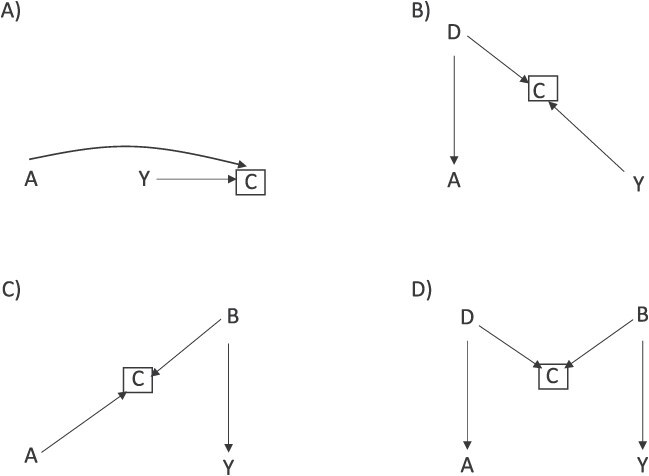
Directed acyclic graphs illustrating 4 common collider stratification bias scenarios. A) Collider C is directly affected by exposure A and outcome Y. B) Collider C is directly affected by exposure A and shares a common cause (B) with outcome Y. C) Collider C is directly affected by outcome Y and shares a common cause (D) with exposure A. D) Collider B is not directly affected by either exposure A or outcome Y, but is affected by common causes of the exposure and outcome (B and D).

**Figure 2:**
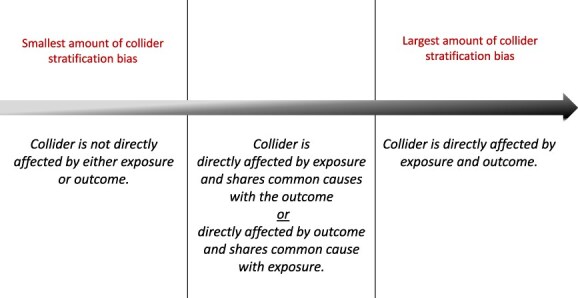
Magnitude of collider stratification bias and relations among the collider, exposure, and outcome.

Nevertheless, there is longstanding debate about the potential magnitude of collider stratification, its impact on study results, and its potential to affect conclusions. Some authors have suggested that the magnitude of bias introduced by collider bias may be quite small in practice but will vary by causal scenario and relations between variables being considered. Figure 1 presents 4 common collider bias scenarios, A-D. Greenland^5^ showed that conditioning on a collider can induce a bias similar in magnitude to confounding when the collider is directly affected by exposure and disease, as depicted in Figure 1A. Smaller degrees of collider bias are expected when conditioning on a variable that is not directly affected by both exposure and outcome, such as in Figure 1B and 1C, and in the case of M bias (Figure 1D). To summarize these concepts: the magnitude of collider stratification bias exists on a continuum (Figure 2). Also, note that the presence or absence of a relationship between exposure and outcome does not influence the magnitude of bias introduced by conditioning on a collider. None of the directed acyclic graphs (DAGs) in Figure 1 include an arrow from exposure to outcome, because the biasing path through collider C is present irrespective of an A-Y arrow; if there was an arrow present, the structure of the bias would remain the same.

Though not easily depicted in DAGs, interaction can substantially affect magnitude of collider stratification bias,^4^^,^^6^ such as between A and B on risk of Y in Figure 1B. A simulation study by Mayeda et al.^4^ in 2018 highlighted the importance of considering interaction between the exposure and common cause of collider and outcome in driving the strength of collider stratification bias. In addition, in causal scenarios with dichotomous variables, prevalence of (1) the collider and (2) common cause of exposure or outcome influences the magnitude of bias introduced. Using Figure 1B as an example, if variable B is very rare, then even strong relations between A-C and B-C will result only small differences in prevalence of B by A and, accordingly, only will introduce a small magnitude of bias. Generally, quantitative bias analysis and simulation studies may be useful to gain insights into potential magnitudes of bias due to collider stratification.^6^^,^^7^

## Bounding approaches to estimate potential collider stratification bias

Bounding approaches can be used to investigate the magnitude of collider stratification bias and can be useful as a tool for sensitivity analysis. Specific to collider stratification, bounds have been derived based on information about the relationships between variables in the causal mechanism to provide investigators estimates of the upper limits of bias introduced in a range of assumptions and scenarios, including M bias and collider-stratification–induced selection bias.^8^^,^^9^

Consider the common collider bias scenario in Figure 1B, wherein exposure directly causes the collider, and there is a common cause of the collider and outcome. As described by Smith and VanderWeele,^8^ there are 4 parameters required to calculate bounds for the amount of bias introduced by conditioning on a collider that can be described as prevalence or risk ratios (RR): the strength of the relationship between B and C in the exposed (A = 1) and unexposed (A = 1): RR_BC|(A = 1)_ and RR_BC|(A = 0)_, respectively; and the strength of the relationship between B and Y in the in the exposed and unexposed: RR_BY|(A = 1)_ and RR_BY|(A = 0)_, respectively. These parameters correspond to the magnitude of the association represented in the arrow from B to C and B to Y, with the understanding that the strength of the relationship may vary in the exposed and unexposed. The parameters can then be used to calculate the bounding factor using the following formula:


(1) \begin{align*} Bounding\ factor&=\left(\frac{{\mathrm{RR}}_{\mathrm{BY}\mid \left(\mathrm{A}=1\right)}\times{\mathrm{RR}}_{\mathrm{BC}\mid \left(\mathrm{A}=1\right)}}{{\mathrm{RR}}_{\mathrm{BY}\mid \left(\mathrm{A}=1\right)}+{\mathrm{RR}}_{\mathrm{BC}\mid \left(\mathrm{A}=1\right)}-1}\right)\ \nonumber\\&\quad\times\ \left(\frac{{\mathrm{RR}}_{\mathrm{BY}\mid \left(\mathrm{A}=0\right)}\times{\mathrm{RR}}_{\mathrm{BC}\mid \left(\mathrm{A}=0\right)}}{{\mathrm{RR}}_{\mathrm{BY}\mid \left(\mathrm{A}=0\right)}+{\mathrm{RR}}_{\mathrm{BC}\mid \left(\mathrm{A}=0\right)}-1}\right) \end{align*}

To quantitatively assess the role of RR_BC|(A = 1)_, RR_BC|(A = 0)_, RR_BY|(A = 1)_, and RR_BY|(A = 0)_ in driving the magnitude of bias, we calculated the bounding factor under varying scenarios when each of the 4 parameters ranged from RR = 1 to RR = 4. This created 256 combinations of bias parameters, each resulting in the calculation of a bounding factor. By varying the 4 parameters in the bounding factor equation, we will gain intuition about what drives the magnitude of collider stratification bias. In the Supplementary Material, we present the results for all possible combinations of these bias parameters; in Table 1 we present some of the scenarios that led to the calculation of a unique bounding factor. Of the 256 possible bounding factor calculations, 50 led to a bounding factor of 1.0, meaning no bias would be introduced under these scenarios. All 50 of these scenarios had an RR equal to 1 for at least 1 of the parameters RR_BC|(A = 1)_, RR_BC|(A = 0)_, RR_BY|(A = 1)_, and RR_BY|(A = 0)_. If the association between either B and C or B and Y are null, no bias is introduced, because S would not be a collider.

**Table 1:** Bounding factors for collider stratification bias.

**Scenario**	**RR**_**BY|(A = 1)**_	**RR**_**BY|(A = 0)**_	**RR**_**BC|(A = 1)**_	**RR**_**BC|(A = 0)**_	**Bounding factor**
1	4	4	4	4	5.2
2	4	3	4	4	4.6
3	4	3	4	3	4.1
4	4	3	3	4	4.0
5	4	2	4	4	3.7
6	4	3	3	3	3.6
7	4	2	4	3	3.4
8	3	3	3	3	3.2
9	2	4	2	4	3.0
10	3	2	3	4	2.9
11	3	2	3	3	2.7
12	4	2	2	4	2.6
13	4	2	2	3	2.4
14	4	1	4	1	2.3
15	4	2	2	2	2.1
16	4	1	3	1	2.0
17	3	1	3	1	1.8
18	4	1	2	1	1.6
19	3	1	2	1	1.5
20	2	1	2	1	1.3
21	1	1	4	4	1.0

Examination of the scenarios demonstrates that RR_BC_ and RR_BY_ are equally important for driving the bounding factor calculation. For example, if RR_BC|(A = 1)_ = 1 and RR_BC|(A = 0)_ = 1, but RR_BY|(A = 1)_ = 4 and RR_BY|(A = 0)_ = 4, the bounding factor = 1.0. Conversely, if RR_BC|(A = 1)_ = 4 and RR_BC|(A = 0)_ = 4, but RR_BY|(A = 1)_ = 1 and RR_BY|(A = 0)_ = 1, the bounding factor is still equal to 1.0. As expected, the largest bounding factor was produced in scenarios with a strong relationship between B and C and B and Y (in particular, when RR_BC_ ≥ 3 and/or RR_BY_ ≥ 3). The greatest bounding factor was calculated in the scenario when RR_BC|(A = 1)_ = 4 and RR_BC|(A = 0)_ = 4, but RR_BY|(A = 1)_ = 4 and RR_BY|(A = 0)_ = 4, resulting in a bounding factor of 5.2. The bounding factor was slightly lower, 4.6, when 1 of RR_BC|(A = 1)_, RR_BC|(A = 0)_, RR_BY|(A = 1)_, or RR_BY|(A = 0)_ was equal to 3 but other parameters were equal to 4. This demonstrates the importance of the strength of each individual parameter in creating the bias.

Smith and VanderWeele^8^ demonstrated mathematically that the magnitude of collider stratification bias affecting an observed AY risk ratio, will be less than or equal to the bounding factor calculated for a specific combination of values for RR_BC|(A = 1)_, RR_BC|(A = 0)_, RR_BY|(A = 1)_, and RR_BY|(A = 0)_, as follows:


(2) \begin{equation*} \left(\frac{\mathrm{Observed}\ {\mathrm{RR}}_{\mathrm{AY}}\ }{\mathrm{True}\ {\mathrm{RR}}_{\mathrm{AY}}}\right)\le \mathrm{Bounding}\ \mathrm{factor} \end{equation*}

A bounding approach is also available to quantify the magnitude of collider stratification bias when exposure and outcome directly affect the collider (Figure 1A) and the M-bias scenario described in Figure 1D. Flanders and Ye^9^ demonstrated empirically that the magnitude of M bias is typically small (<5% bias) but can be larger in scenarios when interaction is present or variables that are highly prevalent (10%-25% bias).

## Conclusions

The structural conditions that produce collider stratification bias are well described in the epidemiologic literature.^1^ However, in practice, the magnitude of the bias is a more important consideration than simply whether the structure is present or absent. Questions about the magnitude of collider stratification bias are longstanding. The answer is a classic epidemiologists’ answer: it depends. As we have described, it depends on the causal structure represented in a DAG, the strength of the relationships between the variables in the causal mechanism, the presence of interaction with the exposure, and the prevalence of the variables being examined. Though, in many instances, the magnitude of bias introduced by conditioning on a collider may be small, in other scenarios, it can cause a reversal of the expected effect. As we have described, bounding approaches may be helpful to estimate the magnitude of bias and for understanding the potential impact of collider stratification in a given study.
